# A Novel Lightweight Human Activity Recognition Method Via L-CTCN

**DOI:** 10.3390/s23249681

**Published:** 2023-12-07

**Authors:** Xue Ding, Zhiwei Li, Jinyang Yu, Weiliang Xie, Xiao Li, Ting Jiang

**Affiliations:** 1 Mobile and Terminal Technology Research Department, China Telecom Research Institute, Beijing 100876, Chinaxiewl@chinatelecom.cn (W.X.);; 2 School of Information and Communication Engineering, Beijing University of Posts and Telecommunications, Beijing 102209, China

**Keywords:** human activity recognition, Wi-Fi sensing, lightweight, L-CTCN

## Abstract

Wi-Fi-based human activity recognition has attracted significant attention. Deep learning methods are widely used to achieve feature representation and activity sensing. While more learnable parameters in the neural networks model lead to richer feature extraction, it results in significant resource consumption, rendering the model unsuitable for lightweight Internet of Things (IoT) devices. Furthermore, the sensing performance heavily relies on the quality and quantity of data, which is a time-consuming and labor-intensive task. Therefore, there is a need to explore methods that reduce the dependence on the quality and quantity of the dataset while ensuring recognition performance and decreasing model complexity to adapt to ubiquitous lightweight IoT devices. In this paper, we propose a novel Lightweight-Complex Temporal Convolution Network (L-CTCN) for human activity recognition. Specifically, this approach effectively combines complex convolution with a Temporal Convolution Network (TCN). Complex convolution can extract richer information from limited raw complex data, reducing the reliance on the quality and quantity of training samples. Based on the designed TCN framework with 1D convolution and residual blocks, the proposed model can achieve lightweight human activity recognition. Extensive experiments verify the effectiveness of the proposed method. We can achieve an average recognition accuracy of 96.6% with only 0.17 M parameter size. This method performs well under conditions of low sampling rates and a low number of subcarriers and samples.

## 1. Introduction

The rapid development of mobile communication and embedded technology has promoted the convergence of communication, computing, multimedia, and other applications, making people’s lives more intelligent [1,2]. The emergence of Internet of Things (IoT) devices provides people with a wide variety of services through various Human–Computer Interaction (HCI) methods, making their lives more convenient [3]. Human activity sensing is a key technology in the field of HCI, where machines provide personalized services to users by analyzing human behavior states and giving corresponding feedback [4].

According to whether users need to carry devices, sensing methods can be divided into device-based [5] and device-free methods [6]. The former mainly use intelligent devices or wearable devices with built-in sensors for perception [7]. The latter mainly leverage video imaging devices or various wireless radio frequency signals for perception [8]. Although the device-based sensing method can achieve great sensing results, it requires users to carry devices throughout the whole perception process, lacking convenience. Although computer vision-based sensing technologies have played a crucial role in intelligent interaction, the perception method has high requirements for image quality and is limited by conditions such as lighting and distance, and it is also constrained by privacy leakage issues. Perception methods based on wireless radio frequency signals can effectively overcome these shortcomings and have gradually gained attention [9,10]. Millimeter-wave [11,12] and ultra-wideband signals [13,14] have strong penetration and detection capabilities and have been widely used in the military, autonomous driving, safety rescue, and other fields. However, specialized signal transceiver devices are costly and challenging to popularize in daily life. In comparison, sensing technology based on wireless communication signals can utilize the widely available basic communication infrastructure, greatly reducing cost [15,16].

With the constant expansion of wireless communication devices, especially low-cost Wi-Fi devices, the coverage of wireless communication networks is expanding, creating favorable conditions for the application of ubiquitous wireless intelligent perception technology [17,18]. Human activity sensing based on Wi-Fi signals is not constrained by lighting conditions, can operate in non-line-of-sight (NLOS) conditions, achieves contactless perception, and effectively protects user privacy. This technology provides an advanced approach to the evolution of intelligent interaction technology [19]. Additionally, the available channel state information (CSI) provides fundamental data support for research on perception technology based on Wi-Fi signals [20]. In summary, human activity recognition technology based on Wi-Fi signals has significant advantages in terms of cost and application deployment [21].

In the field of intelligent perception technology based on Wi-Fi signals, perception methods utilizing deep learning have gained widespread attention due to their efficient automatic feature representation and flexible learning ways [22,23]. Convolutional Neural Networks (CNNs) are widely used in wireless intelligent perception, and this model can effectively learn the local features of the data. WiWrite [24] proposed a CNN-based handwriting recognition system with COTS Wi-Fi. Its properties of local connectivity and weight sharing also reduce the training difficulty of the model, but it lacks memory for sequence data. Subsequently, in order to better utilize the relationship between the time series of wireless signals, Recurrent Neural Networks (RNNs) and Long Short-Term Memory (LSTM) are gradually applied to perception tasks. Refs. [25,26] leverage LSTM to achieve human activity recognition. Recently, researchers explored methods to combine the advantages of the above methods [27,28].

Although research on human activity recognition based on deep learning has achieved great success, complex model structures and a large number of learnable parameters lead to high computational complexity and memory usage. Additionally, perception methods based on deep learning rely on the quality and quantity of data, requiring abundant data samples to fit and train model parameters to ensure model generalization performance [29,30]. Without enough datasets, deep neural networks are prone to overfitting. However, obtaining Wi-Fi activity datasets is a time-consuming and labor-intensive task. Moreover, some methods use high-granularity CSI, increasing data sampling rates and utilizing more subcarriers, which also raises the cost of data processing and storage. The methods that increase the number of parameters, data samples, and data granularity mentioned above bring significant resource consumption while enhancing perception performance. Therefore, the actual deployment of sensing methods still faces significant challenges.

As an important form of interaction, human activity recognition is usually applied to IoT devices. However, the recognition methods based on training complex neural networks with large-scale datasets overlook the computing capabilities and memory resources of IoT devices, making the methods not well-suited for ubiquitous IoT devices. Therefore, it is necessary to explore lightweight, low-cost human activity recognition methods with limited data quality and quantity. This holds significant value for the industrial application of Wi-Fi-based human activity recognition technology.

In this paper, we propose a human activity recognition method based on a Lightweight- Complex Temporal Convolutional Network (L-CTCN). On one hand, the L-CTCN takes complex data as the network input and utilizes complex convolution modules that can fully extract amplitude and phase information from CSI samples. Compared to other networks, the complex network can obtain more action information with the same amount of collected data. On the other hand, the L-CTCN employs a 1D convolution to extract shift-invariant activity features, obtaining temporal features related to actions from CSI samples, which are more relevant to actions. Furthermore, inspired by the TCN [31], MobileNet [32], and MobileNetV2 [33], the design of the TCN framework with residual blocks can significantly reduce the number of model parameters, lowering the network’s computational cost and speeding up the network’s computation speed. We evaluate the performance of the L-CTCN from multiple dimensions through extensive experiments. Our proposed method achieves promising recognition accuracy, effectively decreases the quality and quantity requirements of action samples for the L-CTCN, and reduces the workload of data collection and processing stages, achieving lightweight human activity recognition.

The main contributions of this paper can be summarized as follows:

- We propose a lightweight human activity recognition method, L-CTCN, which leverages a complex network to fully extract activity features, reducing the dependence on the quality and quantity of data samples.
- We design a lightweight model with residual blocks, which reduces the number of model parameters and lowers the computational cost.
- Extensive experiments are conducted to verify the promising performance of this proposed method.

The rest of this paper is organized as follows. Some preliminaries of Wi-Fi sensing are presented in Section 2. In Section 3, the data pre-prepare methods are provided. In Section 4, the proposed L-CTCN is introduced in detail. The experiment results and analysis are given in Section 5. Finally, we conclude this article in Section 6.

## 2. Preliminaries

### 2.1. Channel State Information

Wi-Fi-based human activity recognition utilizes the influence of human action on the propagation of the wireless signal for sensing. CSI can capture the change in the communication link between the transmitter (TX) and the receiver (RX).

The relation between the received signal *y* and transmitted signal *x* can be modeled as

(1) y=Hx+n,

where *n* is the noise vector and $H=H_{R}+iH_{I}$, which is the CSI channel matrix made up of a complex number. For the *s*th subcarrier between the *i*th transmitting antenna and the *j*th receiving antenna:

(2) $$H_{ij}^{s}=∥H_{ij}^{s}∥e^{j∠H_{ij}^{s}},s\in \left[1,N_{s}\right],i\in \left[1,N_{t}\right],j\in \left[1,N_{r}\right],$$

where $∥H_{ij}^{s}∥$ and $∠H_{ij}^{s}$ represent the amplitude and phase, respectively. $N_{t}$ and $N_{r}$ indicate the number of antennas at the TX and RX. And, *i* and *j* are the indices of the TX and RX antennas. $N_{s}$ is the number of subcarriers of each transceiver link.

### 2.2. Data Collection Platform

This paper utilizes the open-source toolkit Linux 802.11n CSI Tools developed by Halperin et al. [34] to set up a Wi-Fi data collection environment that meets the 802.11n wireless protocol. Both the transmitter and receiver are composed of a computer equipped with a commercial Intel 5300 network interface card and three antennas. The computers are running the Ubuntu 12.04 operating system. CSI Tools modify the network card firmware to compute the received packets and output them in a complex CSI form. Both the transmitter and receiver are configured in monitor mode, operating in the 5.32 GHz frequency band with a bandwidth of 20 MHz. The data packet sending interval is set to 5 ms, resulting in a data sending rate of 200 packets (frames) per second. The channel is set to 64, and the transmission mode is set to HT20.

In Wi-Fi wireless communication technology based on 802.11n, Multiple Input Multiple Output (MIMO) and Orthogonal Frequency Division Multiplexing (OFDM) are key technologies to ensure communication capacity and resistance to fading interference. Utilizing OFDM modulation technology, the wireless channel is divided into 64 subchannels, of which 802.11n uses 56 subchannels, with 52 channels for transmitting data signals and 4 channels for transmitting pilot signals. With the CSI Tools toolkit mentioned above, 30 subcarrier data can be obtained. The system we have set up uses three transmit antennas and three receive antennas, allowing us to obtain CSI data for 3 × 3 × 30 = 270 subcarriers.

### 2.3. Dataset Establishing

**Figure 1:**
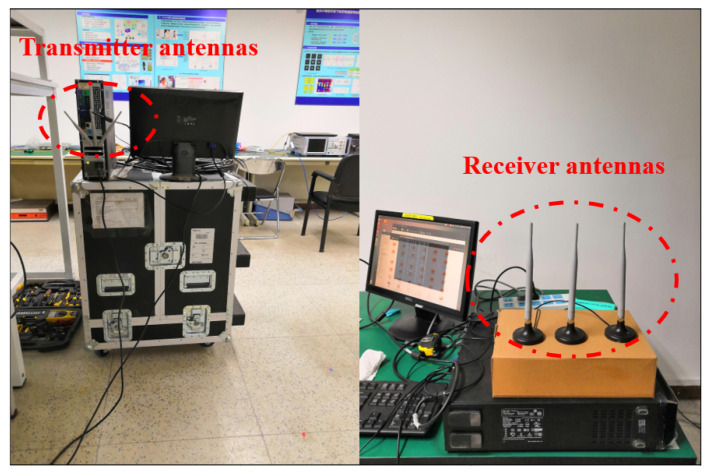
The experimental data collection environment and the transceiver devices.

**Figure 2:**
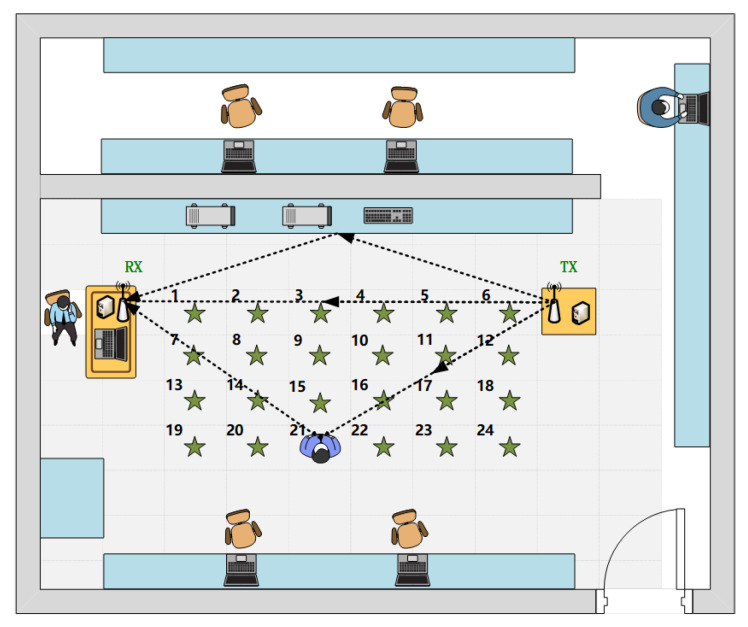
The specific layout of data collection positions.

We collected human activity data samples at 24 positions in an office environment with a size of 6 m × 8 m. The experimental data collection environment and the transceiver devices are shown in Figure 1. The specific layout of the data collection positions is depicted in Figure 2. The distance between the adjacent sampling positions is approximately 0.6 m. The distance between the transmitter and receiver is 4 m. Five predefined human activities include drawing a circle with the right hand (O), drawing a cross with the right hand (X), lifting and lowering both arms (UP), pushing both arms and opening them (PO), and standing up and sitting down (ST). We collected 50 samples for each activity at each sampling position. Thus, there are a total of 24 × 50 = 1200 samples for each activity. During the data collection process, the computer emitted a beep to indicate the start time of collecting each action sample. The beeping interval was 5 s, and each activity sample was completed within 5 s, with the timestamp of the action’s start time recorded.

## 3. Data Pre-Prepare

### 3.1. Data Segmentation

During data collection, the sampling rate of CSI was set to 200 frames per second, and the collection time for a single action was 5 s. With 50 consecutive actions collected each time, we obtained CSI consisting of approximately 50,000 frames. In the subsequent feature extraction, individual samples need to be processed, so data samples need to be segmented. The continuous CSI data is divided into individual CSI action samples based on timestamps. Typically, the actual duration of an action is about 3.5–4 s equivalent to approximately 700–800 frames. Based on the timestamp for the start of each action sample, we extracted 750 frames of data as one action sample. Therefore, for an OFDM-MIMO system with 270 subcarriers, the final collected CSI action sample is a complex matrix of the size 750 × 270.

It is worth noting that data segmentation is very important for processing the collected CSI human activity data. There are many articles that have innovatively proposed data sample segmentation methods [35]. Signal statistical characteristics of amplitude and phase can be used as the segmentation standard. In addition, the subcarrier sequence after signal processing can be also used as the segmentation standard. The former is more likely to be disturbed by noise. The latter is relatively stable. However, in this paper, we did not focus on the instance segmentation method; instead of analyzing signal characteristics to determine whether there is any action, we used computer hardware and recorded time stamps, which means that the starting position of the action is known to us. Therefore, this is equivalent to obtaining a dataset that has already been segmented. However, the instance segmentation method is indispensable for the real-time human activity recognition system. We will explore a novel instance segmentation method to further improve the recognition performance in future work.

### 3.2. Denoising

**Figure 3:**
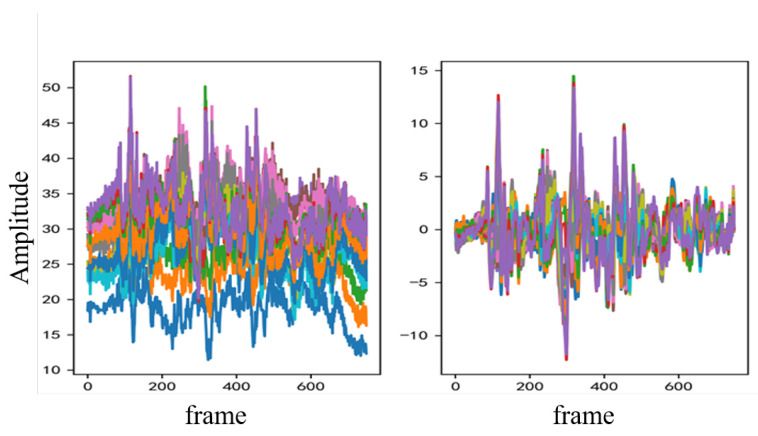
The CSI waveforms before and after band-pass filtering.

The CSI obtained directly from CSI Tools is filled with static components, low-frequency and high-frequency interference, and burst noise. These interferences make it difficult to accurately extract action information, affecting the subsequent recognition effectiveness of the model. Therefore, the action samples need to be denoised first. In our system, the center frequency of the Wi-Fi signal is 5.32 GHz, and based on the formula c=fλ, *c* is the speed of light, and the wavelength of the Wi-Fi signal is approximately 0.05 m. According to the literature [36], when the movement speed of an object is *v*, it results in a maximum fluctuation speed of the wireless signal dynamic path of 2v. Hence, the maximum fluctuating frequency of the Wi-Fi signal caused by human movement is F=2v/λ. Typically, the maximum movement speed of the human body during activity will not exceed 1 m/s, so the maximum fluctuation frequency caused by the human body is 40 Hz. To effectively filter out noise interference from the CSI, this system uses a Butterworth band-pass filter with an upper cutoff frequency of 40 Hz and a lower cutoff frequency of 2 Hz. The CSI waveforms before and after band-pass filtering are shown in Figure 3.

## 4. Human Activity Recognition Method Based on L-CTCN

### 4.1. Motivation

Most existing human activity recognition methods based on deep neural networks primarily utilize either the amplitude or phase information of complex-valued CSI as the network input, converting CSI into real numbers. Additionally, the network parameters are also kept as real numbers, resulting in a very limited utilization of available information. In contrast, complex-valued networks can directly leverage complex data as the network input, where complex data has information in both the real and imaginary dimensions. In other words, for the same data size, complex data provides more information. Therefore, if the network aims to extract equally rich information, fully leveraging complex data can reduce the data size of the network input. Based on this analysis, we explored human activity recognition methods based on complex networks, aiming to maintain recognition accuracy while further reducing reliance on the quality and quantity of the dataset, thereby reducing computational resource consumption during the data processing stage.

On the other hand, the number of learnable parameters in deep learning models correlates with the ability to extract diverse features, but more parameters lead to huge resource consumption, making the model impractical for lightweight IoT devices. In conventional human activity recognition methods, a 2D convolution is generally employed to construct models for feature extraction and recognition. However, models composed of multiple 2D convolutions have an excessive number of learnable parameters, increasing the training cost of the model. In this paper, we need to explore lightweight model methods, reducing the model size to fit ubiquitous IoT devices. It is worth noting that the number of parameters when using 1D convolution will be severely less than that of 2D convolution. Furthermore, for time series problems, RNNs are commonly utilized for modeling due to their recurrent structure, suitable for time sequences. However, RNN-based architectures are computationally expensive in terms of calculation, memory usage, and training. The Temporal Convolutional Network (TCN) [31] has been an effective and lightweight solution for sequence modeling and has demonstrated excellent performance. Compared to the RNN, the TCN has the following advantages: (1) A process in parallel, reducing training and prediction time. (2) Flexible receptive fields, allowing better control of model memory size and adaptation to different tasks. (3) Stable gradients, helping to avoid the vanishing and exploding gradient issues. (4) Lower memory usage.

In this paper, we need to explore lightweight methods, reducing the model size to fit ubiquitous IoT devices. We propose a Lightweight-Complex Temporal Convolutional Network (L-CTCN) for human activity recognition based on complex convolution networks and the TCN framework. To reduce the model’s computational load, we designed the TCN framework. Specifically, we utilized a 1D convolution to extract shift-invariant motion features, capturing temporal features related to CSI samples that are highly correlated with actions. Additionally, inspired by the lightweight MobileNet series architecture [32,33], the TCN framework is designed based on inverted residual blocks, utilizing depth-wise separable convolution to further reduce the network’s computational load. The dilated convolutional kernels are employed to enlarge the receptive field, giving the model a certain degree of interference resistance. This approach significantly reduces the number of model parameters, lowering the computational cost of the network. In the following sections, we will cover the details of this method.

### 4.2. L-CTCN Network Architecture

**Figure 4:**
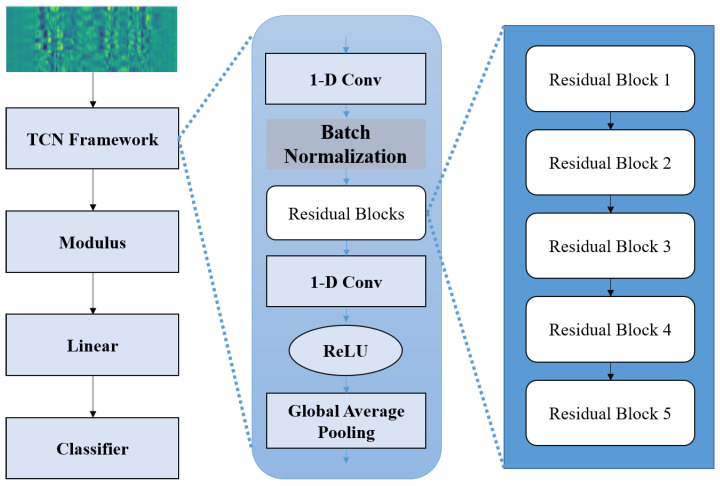
The structure of L-CTCN.

The structure of the L-CTCN is shown in Figure 4. The L-CTCN mainly consists of a TCN framework, which takes complex CSI data as the input. CSI represents the sampled channel frequency response along the time axis. We use the TCN to convolve over the time axis of CSI, extracting features related to time, which can obtain contextual information about the activity. In addition, the L-CTCN utilized complex convolution for feature representation, enabling the thorough extraction of activity-related information from CSI samples.

In order to design the lightweight sensing model, the TCN framework is constructed by 1D convolution and multiple cascaded residual blocks. The residual structure leverages depth-wise separable convolution and dilated convolution, which allows for fewer parameters in the network, enabling lower computational complexity and saving more computational resources without compromising recognition accuracy. In each residual block, the features are first expanded for high-dimensional feature extraction and then compressed, which can reduce the network’s computational cost. After feature extraction using the TCN framework, the features are subjected to modulus operation for input to the classifier for classification. Next, we provide a detailed introduction.

**Figure 5:**
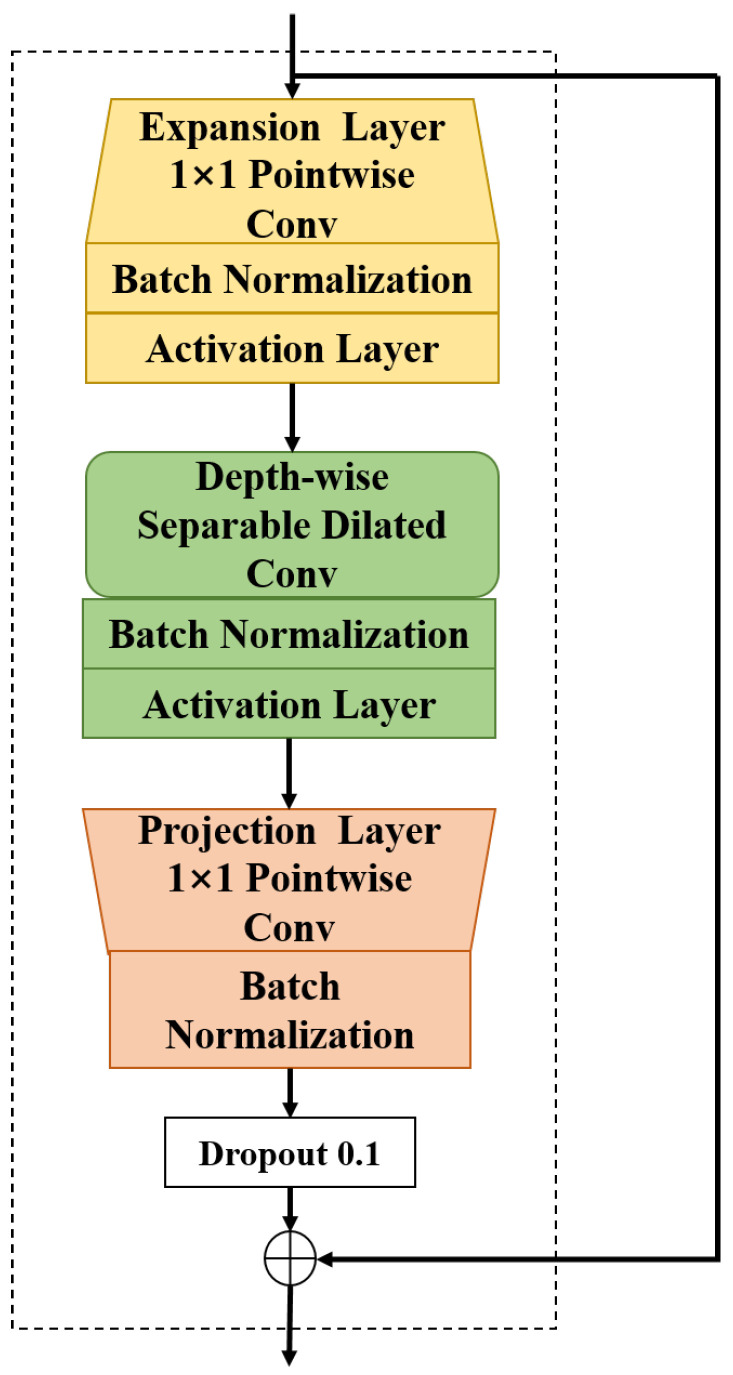
The residual structure.

Considering the number of parameters and the vanishing gradient problem when the network becomes deeper, ResNet has demonstrated that the residual structure is an effective way to train deep networks, allowing the network to transmit information in a skip-layer manner. The TCN is composed of cascaded residual blocks, using residual structures instead of regular convolutional layers. In order to reduce the computing cost while ensuring network performance, the L-CTCN redesigns the residual structure in the original TCN framework. The residual structure consists of residual branching and identity mapping. The residual structure is shown in Figure 5.

The output residual structure can be expressed by the following formula:

(3) o=X+τ(X).

The residual branching contains three transformations:

(4) τ(X)=[E∘N∘P]X,

where *E* is the linear expansion transformation $E:R^{Q\times L}\to R^{2Q\times L}$; *N* is a nonlinear transformation on each channel $N:R^{2Q\times L}\to R^{2Q\times L}$; *P* is a linear projection transformation of the output domain $P:R^{2Q\times L}\to R^{Q\times L}$ indicating the module connection.

In the L-CTCN, *E* and *P* are achieved by point-wise convolution. The convolution layer in the transformation *N* includes depth-wise separable and dilated convolution, and also integrates a weight normalization layer and Hard-Swish (h-swish) activation layer [33]. The Hard-Swish activation function is defined as

(5) h−swish(x)=xReLU6(x+3)6,

where ReLU6=min(6,max(0,x)).

Depth-wise separable convolution, consisting of depth-wise convolution and point-wise convolution, is used to extract feature maps. Compared with conventional convolution operation, the number of parameters and operation costs are lower. One convolutional kernel of depth-wise convolution is responsible for extracting the feature of one channel, which is convolution by only one convolutional kernel. The number of feature map channels generated by this process is the same as the number of input channels. Point-wise convolution aims to combine these feature maps to generate new feature maps. The convolution kernel size is 1 × 1 × M, where M is the number of channels in the previous layer. In this paper, M = 64.

**Figure 6:**
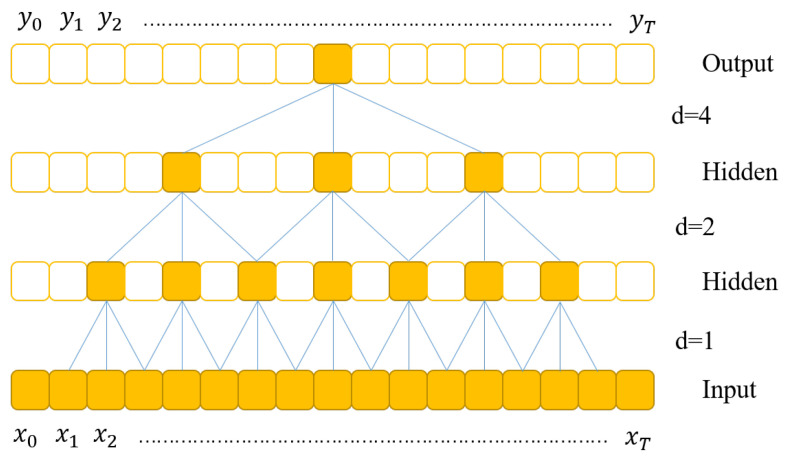
A dilated convolution with dilation factors d = 1, 2, 4.

In traditional deep learning networks, the size of the convolutional kernels is often limited, making it challenging to extract long temporal features and unsuitable for modeling time series problems. To extract a larger range of temporal features, more layers have to be stacked. Dilated convolution can address this issue, increasing the receptive field of the backbone network. For a one-dimensional input sequence $x\in R^{n}$ and a convolutional kernel function $f:\left\{0,\cdots ,k-1\right\}$, the dilated convolution operation F on sequence element s is defined as follows:

(6) $$F\left(s\right)=\left(x{*}_{d}f\right)\left(s\right)=\sum_{i=0}^{k-1}f\left(i\right)\cdot x_{s-d\cdot i},$$

where *d* represents the dilation factor, *r* represents the convolutional kernel size, and *i* represents the convolutional direction. Dilated convolution involves setting a fixed stride between two adjacent convolution operations. When d=1, dilated convolution is equivalent to standard convolution. Using larger dilation factors allows the output of higher-level networks to cover a larger input range, effectively enlarging the receptive field of the network. Thus, in dilated convolution, the network’s receptive field can be increased by increasing the convolutional kernel size *r* and the dilation factor *d*. For instance, the dilated convolution with dilation factors d=1,2,4 is illustrated in Figure 6, where the convolutional kernel size k=3 and the dilation factor *d* are powers of two:

(7) $$d_{b}=2^{b},b=0,1,\cdots ,B.$$

Specifically, in the L-CTCN, the input sample for the TCN architecture is complex CSI data of size 750×15, where 750 represents the length of the input sample, and 15 represents the sample feature count, namely the number of subcarriers. In the TCN framework, the complex data undergoes an initial complex point-wise convolution with a kernel size of one and a stride of one. The number of input channels is 15, and the number of output channels is 32. Subsequently, the intermediate features are inputted into five cascaded residual blocks, each with an input channel of 32 and an output channel of 32. In each residual block, features are first expanded through point-wise convolution, increasing the feature channels from 32 to 64. Then, features are extracted through a depth-wise separable dilated convolutional layer, where the convolutional kernel size is three, the stride is one, and the input and output channels are 64. Next, point-wise convolution is applied to compress the features, reducing the feature channels from 64 to 32. The dilation factors of each residual block is set to 1, 2, 4, 8, and 16, respectively. Following the cascaded residual blocks is another point-wise convolution with both an input and output channel of 32. Finally, the TCN framework ends with global 1D average pooling for feature sampling, followed by a linear layer of size (32, 5) for classification. It is worth noting that the above convolution processes are complex convolution, and the details will be described in the next section.

### 4.3. Complex Convolution Network

The complex convolutional network extends the real-valued convolutional network from the real domain to the complex domain. By combining the feature extraction capabilities of convolutional operation and the abundant information of complex data, the complex convolutional network can provide better feature representation ability. In this paper, the network modules are in a complex form, including the complex convolutional layer, complex activation layer, complex batch normalization layer, complex pooling layer, and complex dropout. These modules are described below in detail.

**Figure 7:**
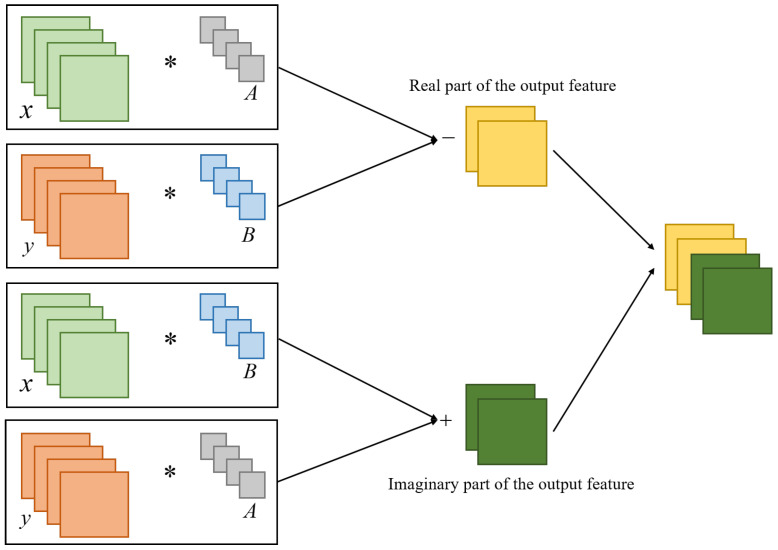
The complex convolution operation.

(1) Complex Convolutional Layer: Assuming the input CSI matrix of the network is P=x+iy, and the complex convolutional kernel is W=A+iB, the process of complex convolution can be represented as follows:

(8) $$\begin{array}{c} W*P=(A+iB)*(x+iy)\\ =(A*x-B*y)+i(B*x+A*y), \end{array}$$

where * represents the convolution operation. This process can be decomposed into four real-valued convolution operations. The matrix form can be expressed as follows:

(9) $$\left[\begin{array}{c} R\left(W^{*}P\right)\\ ℑ\left(W^{*}P\right) \end{array}\right]=\left[\begin{array}{cc} A & -B\\ B & A \end{array}\right]*\left[\begin{array}{c} x\\ y \end{array}\right],$$

where R and *ℑ* represent the real part and imaginary part, respectively. The complex convolution operation is shown in Figure 7.

(2) Complex Activation Layer: The activation function is an indispensable part of deep neural networks, aimed at helping the network learn features from the data. It ultimately determines what content is to be output to the next neuron. In this paper, the activation function CReLU activates the real and imaginary parts of the complex number separately. For a complex input *z*, the output can be expressed as

(10) CReLU(z)=ReLU(ℜ(z))+iReLU(ℑ(z)).

(3) Complex Batch Normalization Layer: Assuming the input complex data is $x=\{x_{1},x_{2},\cdots ,x_{m}\}$, the output of the complex batch normalization can be calculated as

(11) $$\bar{x}=V^{-\frac{1}{2}}\left(x-E\left[x\right]\right),$$

where the expectation *E* is

(12) $$E\left(x\right)=\left[\begin{array}{c} E(ℜ(x))\\ E(ℑ(x)) \end{array}\right]=\left[\begin{array}{c} \frac{1}{m}\sum_{i=0}^{m}ℜ(x_{i})\\ \frac{1}{m}\sum_{i=1}^{m}ℑ(x_{i}) \end{array}\right].$$

*V* is the covariance matrix, calculated as

(13) $$V=\left(\begin{array}{cc} \mathrm{Cov}(ℜ(x),ℜ(x)) & \mathrm{Cov}(ℜ(x),ℑ(x))\\ \mathrm{Cov}(ℑ(x),ℜ(x)) & \mathrm{Cov}(ℑ(x),ℑ(x)) \end{array}\right),$$

where Cov represents the covariance operation. Taking Cov(ℑ(x),ℜ(x)) as an example:

(14) $$\mathrm{Cov}(ℑ(x),ℜ(x))=\frac{\sum_{j=1}^{m}\left(ℑ(x_{j})-E(ℑ(x_{j})\right))\left(ℜ(x_{j})-E(ℜ(x_{j})\right))}{m}.$$

To keep the original feature distribution, scale transformation and translation transformation are conducted [37].

(4) Complex Pooling Layer and Dropout: Pooling and dropout have been widely used in real-valued networks, and pooling layers reduce the dimension of the data features. Complex pooling is based on real-valued pooling. The real and imaginary parts of the complex numbers are pooled separately to reduce data dimension and summarize information. In this paper, a global 1D average pooling convolution layer is used, outputting the average value of each feature channel.

The dropout layer randomly deactivates some neurons to mitigate overfitting during network training. Complex dropout operations are applied separately to the real and imaginary parts of the complex data.

(5) Output Layer Before classification, a modulus operation is performed as the output of the complex network:

(15) $$z^{\left(l+1\right)}=\sqrt{(ℜ{\left(z^{\left(l\right)}\right)}^{2}+(ℑ{\left(z^{\left(l\right)}\right)}^{2}},$$

where $z^{\left(l\right)}$ indicates the output of the *l*th layer of the complex network. Taking the modulus of $z^{\left(l\right)}$ and then inputting the result into the Softmax classifier to recognition the activities.

Similar to real-valued networks, complex networks update the parameters within the network through back-propagation in the training process [37]. The following describes this process in detail.

Assume $a_{k}^{\left(l-1\right)}\in C^{W_{l-1}\times H_{l-1}\times K}$ is the input feature map of the *l* layer in the complex convolutional network, and this convolution layer contains the *J* complex convolution kernel $w_{jk}^{\left(l\right)}\in C^{F\times F\times K\times l}$. Then, the complex convolution result of the input feature map and complex convolution kernel at the *l*th layer is

(16) $$\begin{array}{c} V_{j}^{\left(l\right)}=\sum_{k=1}^{K}w_{jk}^{\left(l\right)}*a_{k}^{\left(l-1\right)}+b_{j}^{\left(l\right)}\\ =\sum_{k=1}^{K}\left(ℜ\left(w_{jk}^{\left(l\right)}\right)*ℜ\left(a_{k}^{\left(l-1\right)}\right)-ℑ\left(w_{jk}^{\left(l\right)}\right)*ℑ\left(a_{jk}^{\left(l-1\right)}\right)+ℜ\left(b_{j}^{\left(l\right)}\right)\right)\\ +i\cdot \sum_{k=1}^{K}\left(ℑ\left(w_{jk}^{\left(l\right)}\right)*ℜ\left(a_{k}^{\left(l-1\right)}\right)+ℜ\left(w_{jk}^{\left(l\right)}\right)*ℑ\left(a_{k}^{\left(l-1\right)}\right)+ℑ\left(b_{j}^{\left(l\right)}\right)\right). \end{array}$$

Suppose that the predicted output of the network is $\overset{⌢}{\;y}$, the true label corresponding to the input is *y*, and the loss function is *L*(*y*,$\overset{⌢}{\;y}$). The deviation of the loss to the parameter is calculated as follows:

(17) $$\begin{array}{c} \frac{\partial L(y,\hat{y})}{\partial w_{jk}^{\left(l\right)}}=\left(\frac{\partial L(y,\hat{y})}{\partial ℜ\left(V_{j}^{\left(l\right)}\right)}\frac{\partial ℜ\left(V_{j}^{\left(l\right)}\right)}{\partial ℜ\left(w_{jk}^{\left(l\right)}\right)}+\frac{\partial L(y,\hat{y})}{\partial ℑ\left(V_{j}^{\left(l\right)}\right)}\frac{\partial ℑ\left(V_{j}^{\left(l\right)}\right)}{\partial ℜ\left(w_{jk}^{\left(l\right)}\right)}\right)\\ +i\cdot \left(\frac{\partial L(y,\hat{y})}{\partial ℜ\left(V_{j}^{\left(l\right)}\right)}\frac{\partial ℜ\left(V_{j}^{\left(l\right)}\right)}{\partial ℑ\left(w_{jk}^{\left(l\right)}\right)}+\frac{\partial L(y,\hat{y})}{\partial ℑ\left(V_{j}^{\left(l\right)}\right)}\frac{\partial ℑ\left(V_{j}^{\left(l\right)}\right)}{\partial ℑ\left(w_{jk}^{\left(l\right)}\right)}\right) \end{array},$$

where $\frac{\partial ℜ\left(V_{j}^{\left(l\right)}\right)}{\partial ℜ\left(w_{jk}^{\left(l\right)}\right)}=ℜ\left(a_{k}^{\left(l-1\right)}\right)$, $\frac{\partial ℜ\left(V_{j}^{\left(l\right)}\right)}{\partial ℑ\left(w_{jk}^{\left(l\right)}\right)}=-ℑ\left(a_{k}^{\left(l-1\right)}\right)$, $\frac{\partial ℑ\left(V_{j}^{\left(l\right)}\right)}{\partial ℜ\left(w_{jk}^{\left(l\right)}\right)}=ℑ\left(a_{k}^{\left(l-1\right)}\right)$, $\frac{\partial ℑ\left(V_{j}^{\left(l\right)}\right)}{\partial ℑ\left(w_{jk}^{\left(l\right)}\right)}=ℜ\left(a_{k}^{\left(l-1\right)}\right)$, then

(18) $$\begin{array}{c} \frac{\partial L(y,\hat{y})}{\partial w_{jk}^{\left(l\right)}}=\left(\frac{\partial L(y,\hat{y})}{\partial ℜ\left(V_{j}^{\left(l\right)}\right)}+i\cdot \frac{\partial L(y,\hat{y})}{\partial ℑ\left(V_{j}^{\left(l\right)}\right)}\right)\left(ℜ\left(a_{k}^{\left(l-1\right)}\right)-i\cdot ℑ\left(a_{k}^{\left(l-1\right)}\right)\right)\\ =-\delta_{j}^{\left(l\right)}{\left(a_{k}^{\left(l-1\right)}\right)}^{*} \end{array},$$

where $\delta_{j}^{\left(l\right)}$ is the error term $\delta_{j}^{\left(l\right)}=-\frac{\partial L(y,\hat{y})}{\partial ℜ\left(V_{j}^{\left(l\right)}\right)}-i\cdot \frac{\partial L(y,\hat{y})}{\partial ℑ\left(V_{j}^{\left(l\right)}\right)}$. The conjugate output of the *k*th neuron in layer l−1 is

(19) $${\left(a_{k}^{\left(l-1\right)}\right)}^{*}=ℜ\left(a_{k}^{\left(l-1\right)}\right)-i\cdot ℑ\left(a_{k}^{\left(l-1\right)}\right).$$

According to the back propagation theory, the weight updating process of the complex network is as follows:

(20) $$w_{jk}^{\left(l\right)}\left(t+1\right)=w_{jk}^{\left(l\right)}\left(t\right)-\eta \frac{\partial L(y,y)}{\partial w_{jk}^{\left(l\right)}\left(t\right)}=w_{jk}^{\left(l\right)}\left(t\right)+\eta \delta_{j}^{\left(l\right)}{\left(a_{k}^{\left(l-1\right)}\right)}^{*}.$$

## 5. Experimental Evaluation

In this section, we evaluate the performance of the proposed L-CTCN-based recognition method in detail, including the experimental settings and performance evaluation.

### 5.1. Experimental Settings

To validate the effectiveness of the proposed method, we divided the collected dataset into three subsets: training set, validation set, and testing set, as shown in Table 1. All 24 locations and five actions were utilized for each subset. In the training set, 10 samples were selected for each action from each location, resulting in a total of 1200 samples for training. The number of validation set samples was the same as the training set. For the testing set, the remaining 30 samples were used to evaluate the model, resulting in 720 samples for each action at 24 locations.

The L-CTCN model proposed in this paper was implemented using the PyTorch framework. The batch size was set to 16, and the model was trained for 100 epochs. Stochastic Gradient Descent (SGD) was used for optimization during the training phase, with an initial learning rate of 0.001. The learning rate was adjusted using the “ReduceLROnPlateau” strategy: if the training loss did not decrease for four consecutive epochs, the learning rate would be halved. The loss function was the cross-entropy loss. The average of the test results from the last 10 epochs after the model converged was taken as the recognition result.

In the TCN network used for comparison in this paper, a total of five residual structures were stacked, each consisting of a convolutional layer with a kernel size of three and a stride of one. The parameters for the convolutional layers in the residual structure were set as follows: The number of convolutional kernels in each of the five residual structures was 32, 128, 256, 128, and 32, respectively. The last residual structure outputted 32 features, which were then sampled using a global 1D average pooling layer. Finally, a linear layer of size (32, 5) was used for classification.

### 5.2. Performance Evaluation

#### 5.2.1. Overall Performance of L-CTCN

**Figure 8:**
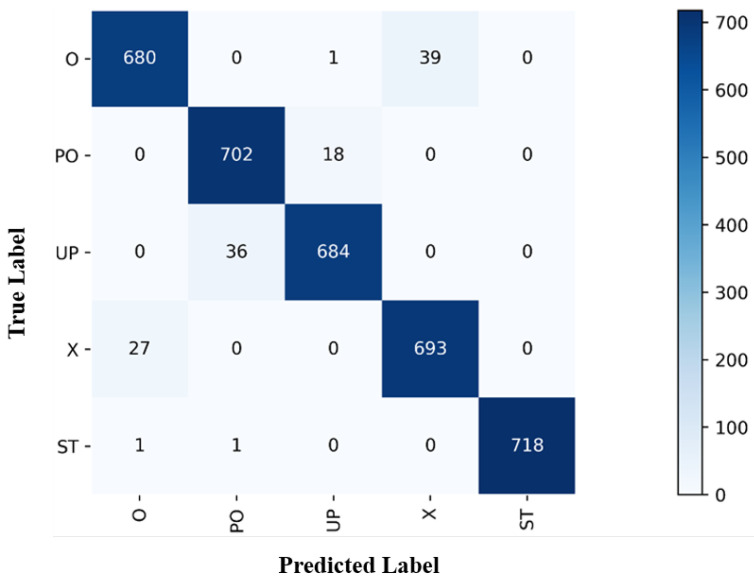
The confusion matrix of L-CTCN.

In this section, the first 15 subcarriers out of the 270 subcarriers in the CSI data are selected as the sample data. Figure 8 shows the recognition results of the L-CTCN, achieving an average recognition accuracy of 96.6% across 24 locations and five activities. In 720 samples of testing data for each action, the numbers of accurately predicted samples for actions O, PO, UP, X, and ST are 680, 702, 684, 693, and 718, respectively. To sum up, the L-CTCN directly uses complex CSI as the input, allowing the network to more effectively extract information, resulting in a high recognition accuracy.

#### 5.2.2. Comparison with Other Methods

To demonstrate the superiority of the L-CTCN, we compared it with other popular state-of-the-art network architectures widely used in human activity recognition, including Bi-LSTM and MobileNetV2. It is important to note that all networks for comparison were trained and then tested on the same test dataset. We evaluated the models in terms of recognition accuracy, parameter size (Params), and the Multiply–Accumulate Operations (MACs). The experimental results are shown in Table 2, where the L-CTCN outperforms the baseline TCN model by 4.3% in recognition accuracy, with only 15% of the computational cost of the TCN. The L-CTCN significantly outperforms Bi-LSTM and MobileNetV2 by 16.7% and 10.5% in terms of recognition accuracy, respectively.

#### 5.2.3. Influence of Different Training Sample Sizes

**Figure 9:**
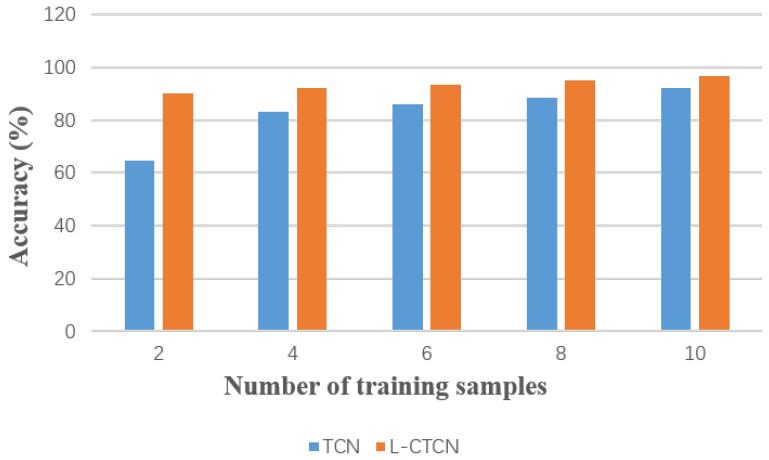
Influence of different training sample numbers on the performance of L-CTCN.

Since collecting sufficient samples in multilocation human activity recognition is a labor-intensive task, it is necessary to study the performance of the model in the case of fewer samples. In this experiment, we discuss the situations involving when the training sample size for each location and action was set to 10, 8, 6, 4, and 2. The experimental results are shown in Figure 9. Even with only two training samples for each action at each location, the L-CTCN still achieves an accuracy of 90.1%. As the number of training samples decreases, the performance gap between the L-CTCN gradually widens. This may be because the TCN contains too many learnable parameters and is prone to overfitting with a small number of samples.

#### 5.2.4. Influence of Different Subcarrier Numbers and Sampling Rates

**Figure 10:**
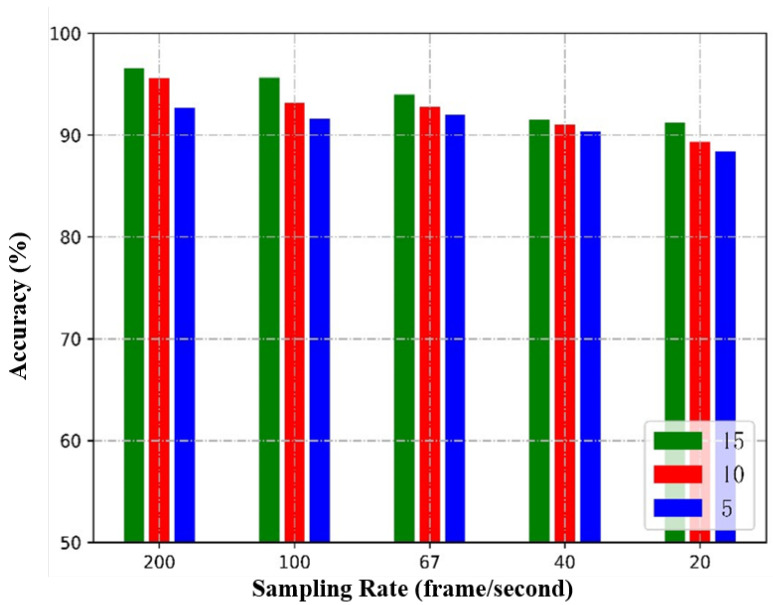
Influence of different numbers of subcarrier and sampling rates on the performance of L-CTCN.

This experiment investigated the impact of low-resolution data on the performance of the L-CTCN. During data collection, the initial sampling rate of CSI was 200 packets per second. To study the effect of low-resolution data on the model, the sample sampling rate was reduced from 200 to 100, 67, 40, and 20 through down-sampling. Additionally, the influence of different subcarrier numbers on the performance of the model was also studied, starting with 15 subcarriers and then gradually decreasing to 10 and 5. The experimental results under different subcarrier numbers and sampling rates are shown in Figure 10. It can be observed that when the sampling rate is 20 and the subcarrier number is five, the L-CTCN still achieves an accuracy of 88.4%. In this case, the TCN has an accuracy of 69.8%.

#### 5.2.5. Ablation Study of L-CTCN

To demonstrate the effectiveness of the proposed L-CTCN, we performed ablation experiments. The experimental results are shown in Table 3. The effects of several parts are discussed, including complex convolution, dilated convolution, and residual blocks. The experimental results are shown in Table 1. When complex convolution is removed, the recognition accuracy decreases by 1.4%. When the dilated convolution is removed, the recognition result decreases by 0.5%. When the inverted residual blocks are removed, and both the input and output channels inside the residual structure are set to 64, the result decreases by about 0.7%. When both the complex convolution and the inverted residual blocks are removed, the real TCN results in a 4.3% drop. It can be seen from the experimental results that each module contributes to improving the recognition accuracy, and complex convolution has the most obvious improvement in network performance.

#### 5.2.6. Comparison Study in Four Different Location Areas

**Figure 11:**
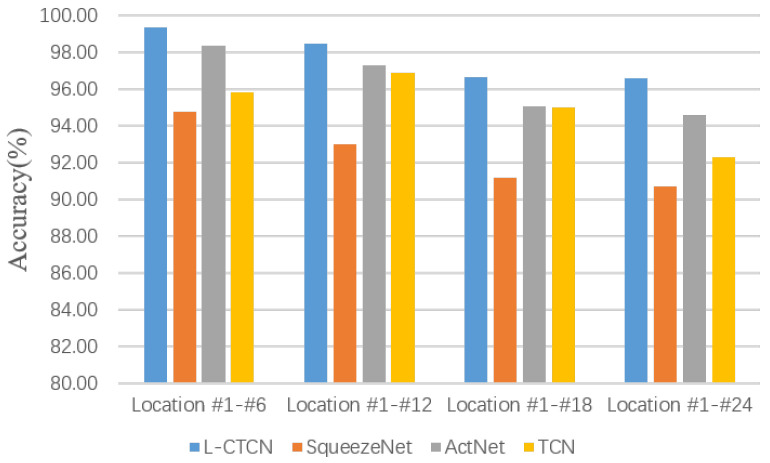
Comparison study in four different location areas.

In this section, deep learning-based human activity recognition methods are conducted to prove the effectiveness of the L-CTCN model for human activity recognition. In the experiments, we compared the L-CTCN with the classical deep learning methods [31,39], and deep learning-based human activity recognition method [29]. We also evaluated the method in different location areas. The experimental results are shown in Figure 11. As we can see, the proposed L-CTCN has obvious performance improvement compared with the other three methods.

## 6. Conclusions

In this paper, a Lightweight-Complex Time Convolutional Network, L-CTCN, was designed for human activity recognition based on Wi-Fi signals. This method effectively alleviates the dependence of the human activity recognition algorithm on the quantity and quality of data samples and reduces the workload in the data acquisition stage and the calculation cost in the data processing stage. The L-CTCN can be deployed in lightweight Internet of Things devices, promoting the process of the industrial application of human activity recognition. On the one hand, the L-CTCN uses a complex convolution module to fully extract the amplitude and phase information in CSI samples. On the other hand, the L-CTCN designed a model architecture based on a residual structure, which can greatly reduce the number of parameters and calculation overhead of the network. Extensive experiments verified the effectiveness and superiority of this approach. As with the other deep learning-based human activity recognition methods, the L-CTCN is still limited by the number of training locations. In addition, instance segmentation is very important for processing the collected CSI human activity data, which is indispensable for the real-time system. In future work, we will explore this problem further.

## Figures and Tables

**Table 1:** Dataset.

Subset	Training Set	Validation Set	Testing Set
Location	24	24	24
Activity	5	5	5
Number of samples	10	10	30

**Table 2:** Comparison of different methods.

Methods	Accuracy	Params (M)	MACs (G)
Bi-LSTM [38]	79.9%	0.2	0.1
MobileNetV2 [33]	86.1%	2.2	0.2
TCN [31]	92.3%	1.1	1.7
L-CTCN	96.6%	0.2	0.3

**Table 3:** Ablation study of L-CTCN.

Methods	Average Accuracy
TCN	92.3%
L-CTCN without complex convolution	95.2%
L-CTCN without inverted residual blocks	95.9%
L-CTCN without dilated convolution	96.1%
L-CTCN (ours)	96.6%

## Data Availability

Data sharing is not applicable to this article.
